# Management of rTTS associated with PGL using VA-ECMO and surgical resection: a case report

**DOI:** 10.3389/fcvm.2024.1468415

**Published:** 2024-09-30

**Authors:** Kechun Zhou, Guoliang Liu, Yi Chen, Li Lin, Pin Lan

**Affiliations:** ^1^ Department of Emergency Medicine, The Fifth Affiliated Hospital of Wenzhou Medical University, Lishui Central Hospital, Lishui Hospital of Zhejiang University, Lishui, Zhejiang, China; ^2^ Department of Emergency Medicine, People's Hospital of Lishui Liandu District, Lishui, Zhejiang, China

**Keywords:** reverse Takotsubo cardiomyopathy, hypertension, paraganglioma, echocardiography, ECMO, surgery

## Abstract

**Background:**

Paraganglioma (PGL) is a neuroendocrine tumor located outside the adrenal gland that can secrete catecholamines. Clinical manifestations include headaches, hypertension, and, rarely, cardiomyopathy. Among these, reverse Takotsubo cardiomyopathy (rTTS) is a rare Takotsubo cardiomyopathy (TTS) associated with a surge in catecholamines.

**Case introduction:**

This article reports a case of a hypertensive patient admitted for recurrent dizziness and chest tightness. During treatment, the patient suddenly experienced chest tightness and shortness of breath, followed by refractory cardiogenic shock, and was eventually diagnosed with rTTS. The patient gradually recovered and was successfully discharged after receiving treatments, including tracheal intubation with mechanical ventilation, extracorporeal membrane oxygenation (ECMO), and surgery.

**Conclusion:**

The diagnosis of rTTS is significantly aided by the presence of free plasma metanephrines and specific changes observed in cardiac ultrasound. In the treatment of severe rTTS, ECMO can serve as a crucial life support technology. Under VA-ECMO support, early resection of the PGL after accelerated preoperative preparation may be a feasible approach.

## Introduction

Pheochromocytoma (PHEO) and PGL are rare neuroendocrine tumors with an incidence of approximately 0.04–0.95 cases per million people (1). PGL can be classified into two subtypes: parasympathetic and sympathetic. Sympathetic PGL typically originate from chromaffin cells in the sympathetic ganglia chain of the thorax and abdomen. These cells can produce catecholamines, leading to clinical symptoms such as paroxysmal or persistent hypertension, dizziness, headache, palpitations, and excessive sweating. When the tumor is subjected to external pressure or other stimuli, it may suddenly release large amounts of catecholamines, causing acute pulmonary edema, cardiomyopathy, and heart failure. Catecholamine-induced cardiomyopathy is uncommon, but its incidence in PHEO and PGL patients is about 8%–11%, with PGL patients accounting for approximately 10% (2).

TTS patients exhibit transient left ventricular dysfunction, characterized by apical ballooning or mid, basal, or focal wall motion abnormalities. Studies have shown that 81.7% of TTS cases occur in the apical segment, whereas rTTS is the rarest type, accounting for only 2.2% (3). In patients with TTS experiencing cardiogenic shock, inotropic agents may be less effective and could potentially worsen wall motion abnormalities. Mechanical methods are recommended for providing hemodynamic support, such as intra-aortic balloon pump, temporary left ventricular assist devices, and ECMO (4). In this report, we present a case of a middle-aged female who was admitted with recurrent dizziness and chest tightness. She developed refractory cardiogenic shock, was diagnosed with rTTS, and received VA-ECMO support. Subsequently, with VA-ECMO maintenance and short-term *α*-adrenergic receptor blocker use, the patient successfully underwent emergency PGL resection and recovered well to be discharged.

## Case report

**Figure 1:**
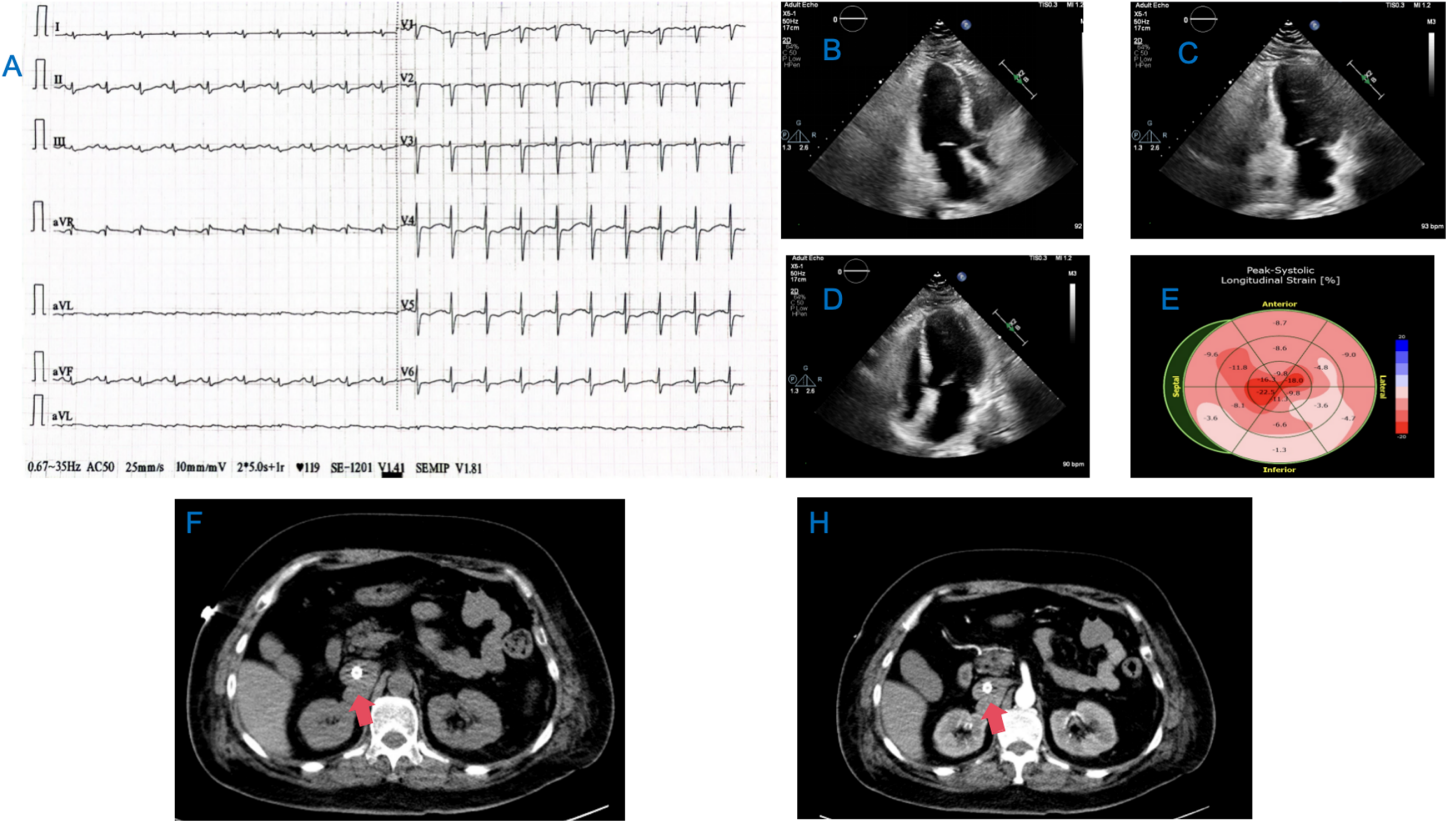
Results of the patient's ECG, cardiac ultrasound, and abdominal CT. **(A)** Sinus rhythm with ST segment depression and T wave inversion in leads V3-V6; **(B**–**D)** Ultrasound images of the patient's heart in the 2-chamber, 3-chamber, and 4-chamber views. **(E)** Graph of the Peak-Systolic Longitudinal Strain of the heart; **(F,H)** CT images of the patient's abdomen, with the red arrows indicating the PGL.

This case report presents a middle-aged female patient with a history of hypertension (long-term use of amlodipine tablets), admitted to our hospital for treatment of recurrent dizziness and chest tightness lasting over two years. Two years ago, the patient experienced dizziness with chest tightness and palpitations without any obvious triggers, and no symptoms of nausea, vomiting, chills, fever, or chest pain. She had visited local hospitals multiple times, undergoing coronary angiography, cardiac ultrasound, and cranial MRI, and was ultimately diagnosed with hypertensive heart disease. Seeking further treatment, the patient came to our hospital. After admission, the patient again experienced palpitations and chest tightness. An ECG was performed (Figure 1A) along with relevant blood tests (BNP 8.0 pg/ml, procalcitonin 0.04 ng/ml, ALT 52 U/L, glucose 24.29 mmol/L, potassium 2.63 mmol/L, creatine kinase 98 U/L, CK-MB 0.53 ng/ml, myoglobin 36.9 ng/ml, troponin <0.012 ng/ml). The patient was treated with potassium supplementation, spironolactone for diuresis, nitroglycerin for vasodilation, and insulin for blood glucose control. However, the patient's chest tightness worsened, her blood oxygen saturation decreased, and she started coughing up pink frothy sputum. She was urgently intubated and placed on mechanical ventilation, then transferred to the intensive care unit (ICU) for further treatment.

In the ICU, follow-up blood tests showed: CBC: rapid hs-CRP 0.66 mg/L, WBC 9.51 × 10^9^/L, RBC 4.97 × 10^12^/L, arterial blood gas: pH 7.047, CO2 partial pressure 33.8 mmHg, oxygen partial pressure 53.1 mmHg, base excess −22.3 mmol/L, blood glucose (whole blood) 41.00 mmol/L, whole blood lactate 13.5 mmol/L; myocardial enzyme profile: creatine kinase 217 U/L, CK-MB 6.96 ng/ml, myoglobin 1,746.0 ng/ml, troponin 1.780 ng/ml, brain natriuretic peptide 273.0 pg/ml. Cardiac ultrasound indicated: left ventricular systolic dysfunction, significantly reduced basal activity, left atrial enlargement, EDV 98.41 ml, ESV 75.77 ml, LVEF 23% (Figures 1B–E). After a cardiology consultation, the diagnosis was considered acute left heart failure.

**Figure 2:**
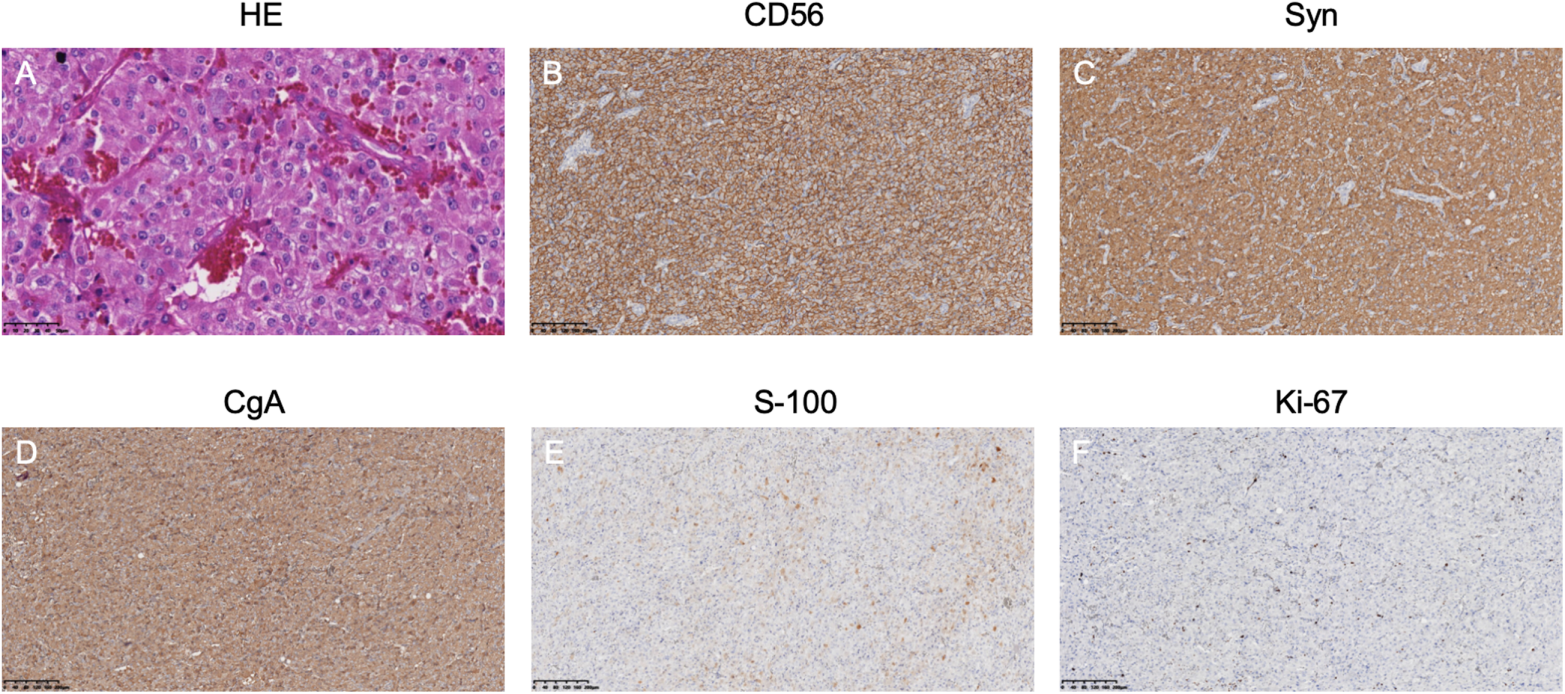
Athology results of the right retroperitoneal mass: **(A)** HE staining shows a nested arrangement of tumor cells. **(B–D)** Immunohistochemistry indicates positive neuroendocrine markers. **(E)** S-100 staining shows supporting cells. **(F)** Ki-67 labeling index is 5%.

**Figure 3:**
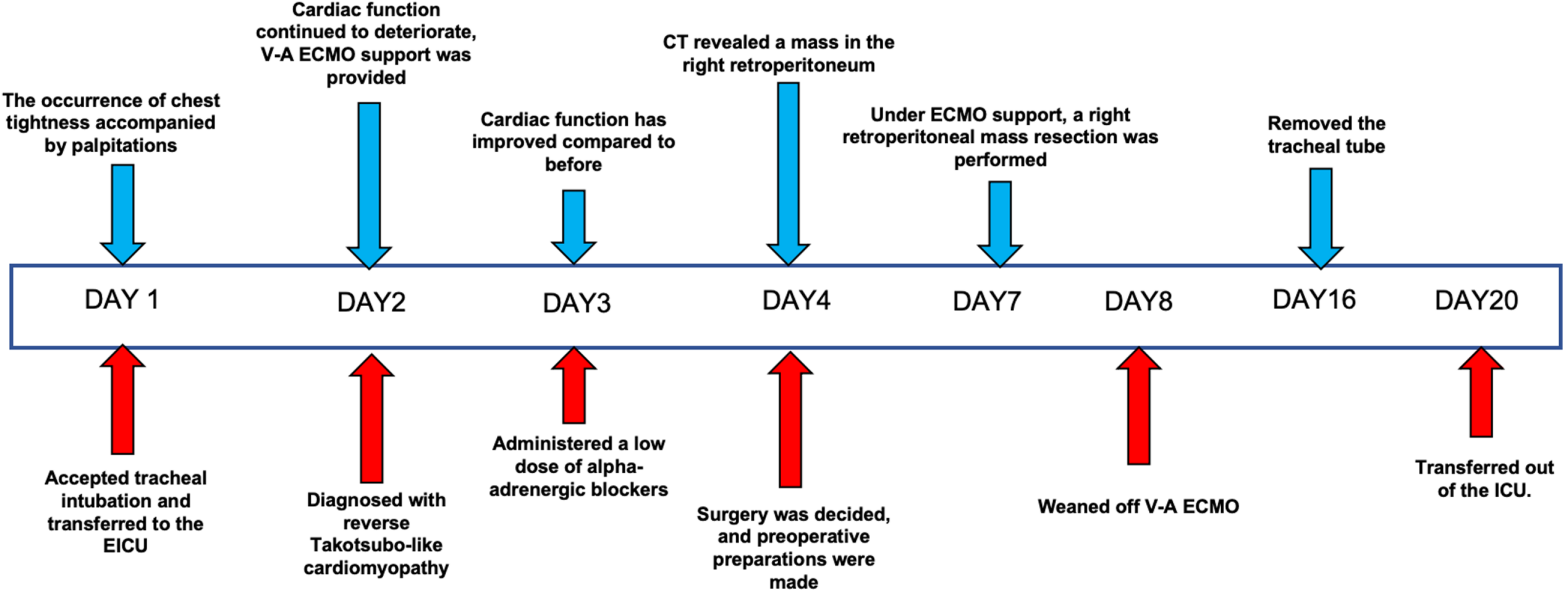
Patient's treatment course.

On the second day of admission, the patient's cardiac function progressively declined, leading to refractory cardiogenic shock, requiring high doses of inotropic drugs for maintenance, and frequent ventricular fibrillation. With family consent, V-A ECMO treatment was initiated. After successful ECMO initiation, the norepinephrine dose was gradually reduced and discontinued. Due to myocardial stunning post-ECMO, a small dose of epinephrine at 0.13 µg/kg/min was maintained. Catecholamine test results from the previous day (without any vasopressor drugs) (Table 1) showed: normetanephrine 1.87 nmol/L, metanephrine >10.14 nmol/L. Combined with the ECG, cardiac ultrasound, and coronary angiography results, we diagnosed the patient with rTTS due to abnormal catecholamine release. On the third day of admission, the patient's cardiac function showed some recovery, with no further increase in myocardial enzymes, and vasopressor drugs were discontinued. We then administered phentolamine at 0.2 mg/min to dilate blood vessels and reduce cardiac load. On the fourth day of admission, the patient was taken for a CT scan, which unexpectedly revealed a mass in the right retroperitoneal area (Figures 1F,H). After multidisciplinary discussions (MDT), we considered that the patient's recurrent dizziness and chest tightness over the past two years were due to abnormal catecholamine release, likely caused by the retroperitoneal mass. With family consent, right retroperitoneal mass resection was performed under ECMO support. Preoperatively, the patient was given adequate fluid resuscitation and gradually increased doses of alpha-adrenergic blockers. Considering the ECMO maintenance and the need for routine heparin anticoagulation, the high risk of intraoperative bleeding led to the decision to pause heparin 12 h before surgery and reassess coagulation function, showing an APTT of 24.6 s. On the seventh day of admission, right retroperitoneal mass resection was performed under ECMO support. Despite severe intraoperative bleeding, symptomatic treatment ensured a successful surgery. Pathology results indicated PGL (Figure 2). Postoperatively, the patient's vital signs were stable without PPGL crisis, and cardiac function recovered well, leading to ECMO removal on the eighth day of admission. After a period of ventilator training and symptomatic supportive treatment, the patient improved, and the tracheal tube was removed on the sixteenth day of admission. Finally, on the twentieth day of admission, the patient was transferred out of the ICU and successfully discharged after a series of rehabilitation treatments in the general ward. Figure 3 is a timeline of the clinical condition progress and major management of the patient. Table 1 presents the patient's levels of methoxamine, metaraminol, myoglobin, troponin, and BNP before admission, during hospitalization, and after the resection of the right retroperitoneal mass.

**Table 1:** The patient's blood test results table.

Project	Metaraminol (nmol/L)	Methoxamine (nmol/L)	Myoglobin (ng/ml)	Troponin (ng/nl)	BNP (pg/ml)
First day	>10.14	1.87	1,746	1.78	83
Second day	>10.14	5.96	>2,000	13.3	1,816
Third day	6.86	>10.92	406.9	10.3	1,271
Fourth day	4.28	7.74	119.5	4.36	702
Fifth day	3.48	5.67	99.8	1.94	496
Sixth day	2.17	3.58	186	1.41	393
Seventh day (after surgical)	3.48	5.67	881.8	0.678	1,091
Eighth day	0.12	0.47	704	0.786	528

## Discussion

rTTS is a rare but severe TTS typically caused by a surge in catecholamines, leading to a sharp rise in blood pressure and acute heart failure. Due to the low incidence of hypertension induced by catecholamines, it is often overlooked. When TTS occurs, it can severely threaten the patient's life. A study found that the mortality rate for patients with TTS during the acute hospitalization period is 4.2% (3). Early diagnosis and treatment are therefore crucial. According to a study, measuring plasma free metanephrine shows high sensitivity and specificity in diagnosing PGL (5). However, most severe patients with TTS use epinephrine or norepinephrine, resulting in elevated plasma metanephrine levels, which can affect the diagnosis. In this case, because we collected blood samples when the patient experienced chest tightness, it helped in the later diagnosis. Cardiac ultrasound also plays an important role in diagnosing rTTS (6). Typical features of TTS include transient systolic and diastolic dysfunction with various wall motion abnormalities, classified into four types: Apical Type, Mid-Ventricular Type, Basal (Reverse) Type, and Focal Type. As coronary artery disease was excluded in this patient, we ultimately diagnosed rTTS based on the International Takotsubo Diagnostic Criteria (7), and later discovered a PGL, explaining the abnormal catecholamine release.

The latest mechanisms regarding catecholamine-induced TTS include: adrenaline surge, myocardial reaction, myocardial toxicity, and neuro-cardiac axis (8). Due to these mechanisms, inotropic drugs may further deteriorate LV wall motion, and experts consistently recommend mechanical methods to provide hemodynamic support, such as ECMO (9). In this case, the patient's cardiac function deteriorated rapidly, and severe hypotensive shock occurred. When conventional inotropic drugs were ineffective, we quickly initiated the ECMO team, providing early V-A ECMO support to restore cardiac function promptly. Within 24 h of successful ECMO operation, the patient's myocardial enzymes no longer increased, and significant improvement in cardiac function was observed within 48 h.

As the patient's cardiac function continued to improve, we faced a new challenge: the timing of surgery. A recent article on the perioperative management of pheochromocytoma and sympathetic ganglioma suggests starting alpha-adrenergic receptor blockers at least 14 days before surgery to reduce surgical mortality and complication rates (10). Since the 1960s, the preoperative use of alpha-blockers (e.g., phenoxybenzamine) to control blood pressure and prevent intraoperative hemodynamic instability and cardiovascular events has been standard practice. With the advent of new techniques such as laparoscopic surgery, the mortality and complication rates of pheochromocytoma surgery have significantly decreased, but the preoperative preparation time has not changed. A systematic review found that preoperative use of alpha-blockers did not significantly improve intraoperative hemodynamic stability or reduce postoperative complications; the incidence of intraoperative hypertension and hypotension events did not differ significantly regardless of alpha-blocker use (11). After discussion among anesthesiologists, endocrinologists, cardiologists, and urologists, it was decided to perform laparoscopic surgery on the fifth day of alpha-adrenergic receptor blocker use. Due to the need for heparin anticoagulation during ECMO operation, which increases surgical risk, heparin was paused 12 h before surgery. Heparin's half-life in normal adults is 1–2 h, with only 0.002%–1.56% remaining in the body after 12 h. Despite concerns about potential risks without heparin, studies indicate that heparin-free VA-ECMO support does not increase the risk of mortality, pump failure, or thromboembolic complications (12). Although we paused heparin use in advance and routinely monitored prothrombin and thromboelastography, intraoperative bleeding was still more significant than in conventional surgery. After active treatment, the surgery was successfully completed without severe complications, and ECMO was successfully removed the next day. This successful experience provides valuable insight for treating similar patients in the future.

## Conclusion

rTTS can be diagnosed by the level of plasma free metanephrine and the specific changes observed in cardiac ultrasound. Catecholamine-induced rTTS appears to be reverse and can be cured with timely and appropriate treatment. When severe cardiogenic shock occurs, ECMO is a good option. Under VA-ECMO support, short-term use of alpha-adrenergic receptor blockers and early resection of the PGL might be a feasible treatment plan.

## Data Availability

The original contributions presented in the study are included in the article/Supplementary Material, further inquiries can be directed to the corresponding author.
